# Comparing the Difference in Traction Between the Bare Hoof, Iron Horseshoes and Two Glue-On Models on Different Surfaces

**DOI:** 10.3390/s25195975

**Published:** 2025-09-26

**Authors:** Claudia Siedler, Yuri Marie Zinkanel, Johannes P. Schramel, Christian Peham

**Affiliations:** 1 Department for Small Animals and Horses, Centre for Equine Health and Research, University of Veterinary Medicine Vienna, 1210 Vienna, Austria; 2 Equine Clinics, Movement Science Group, University of Veterinary Medicine Vienna, 1210 Vienna, Austria; johannes.schramel@vetmeduni.ac.at

**Keywords:** grip test, hoof, horseshoes, rotational resistance, friction

## Abstract

The interaction between equine hooves and various ground surfaces is a critical factor for injury prevention and performance in modern equestrian sports. Accurate measurement of surface grip is essential for evaluating the effectiveness of different hoof protection systems. This study introduces the Vienna Grip Tester (VGT), a novel sensor-based device developed to quantify rotational resistance—an important parameter for assessing hoof–surface interaction. The VGT utilizes a torque wrench and spring-loaded mechanism to simulate lateral hoof movements under a standardized vertical load (~700 N), enabling objective grip measurements across different conditions. Twenty combinations of hoof protection (barefoot, traditional iron shoe, and two glue-on models) and surfaces (sand, sand with fiber at 25 °C and −18 °C, frozen sand, and turf) were tested, yielding 305 torque measurements. Statistical analysis (repeated-measures ANOVA with Bonferroni correction) revealed significant differences in grip among surface types and hoof protection systems. Frozen surfaces (SDAF (31 ± 8.9 Nm and SDF 33 ± 8.7 Nm, *p* < 0.001) exhibited the highest grip, while dry sand (SDA (18.3 ± 3.3 Nm, *p* < 0.001) showed the lowest. Glue-on shoes (glue-on grip, 26 ± 10 Nm; glue-on, 25 ± 10 Nm) consistently provided superior grip compared to traditional or unshod hooves (bare hoof, 21 ± 7 Nm). These results validate the VGT as a reliable and practical tool for measuring hoof–surface grip, with potential applications in injury prevention, hoof protection development, and surface optimization in equestrian sports.

## 1. Introduction

Since the domestication of horses, humans have sought ways to protect their feet on surfaces differing from their natural grassland habitats. Early hoof protection was essential to maintaining horses for work, transport, and warfare [1].

Today, horses are mainly used in sports and leisure activities. This has led to a demand for hoof protection that is affordable, durable, easy to apply, and ensures good ground interaction. While iron horseshoes remain common, modern alternatives such as glue-on shoes and hoof boots offer improved cushioning and surface interaction [2].

The various ground surfaces that a horse has to navigate in its daily life in a regular stable facility make a strong grip a necessity. Ensuring optimal hoof grip on equestrian sport surfaces is a fundamental requirement for the safety and performance of both riders and horses. Previous studies suggest a direct correlation between the amount of sliding on the ground and resulting injuries, which often affect the tendons of the lower limb [3]. Grip measurements that evaluate the grip of a surface and shoe combination are necessary to guaranteeing safe equestrian sports.

There are various methods for measuring slip resistance (grip). A common method is to determine the coefficient of friction. This involves measuring the horizontal force applied to a surface that is required to initiate movement. The coefficient of friction is the ratio of the horizontal force to the vertical force (weight). A higher value indicates better grip. Another approach is to measure torsional resistance, which is the resistance to rotational movements and is particularly relevant for the dynamic movements of a horse [4].

For sports floors in multi-purpose sports halls, the central standard is defined in [5]. This standard specifies various requirements for sports floors, including their sliding properties. Our group developed the “Vienna Grip Tester” (VGT), a device designed specifically for equestrian surfaces. It is based on torsional resistance measurement. VGT measurements record the resistance to rotational movements, which provides information about the hoof’s ability to stabilize itself on the ground during lateral or rotational movements, thereby providing a direct statement about the grip [6,7].

With a variety of surfaces, from indoor concrete floors to the asphalt coatings of loading and parking areas and competition grounds made of sand, mud, turf, and, depending on the season, ice, traditional shoeing methods are challenged. This paves the way for the development of alternatives that are better suited to ground conditions and promote the health of the horses while working.

Regarding the impact on performance, the horse has to adjust the force applied to the limb to propel it forward when moving through competition courses. This is in direct correlation with the grip provided when interacting with the ground, as the horse has to adjust the applied force and the directly related speed to avoid slipping, which is a hinderance during competition [8]. Previous studies analyzed the effects of variable grip in different shoeing options and on various grounds indirectly, for example, by measuring the displacement of the jockey’s center of mass during a gallop [9].

The goal of this study was to measure rotational resistance using the VGT, thus demonstrating the grip of 20 different surface–shoe combinations. This study is necessary to enhance the safety of riders and sport horses and to help prevent injuries. Using our device, hoof–surface grip can be quantitatively assessed, allowing for a better evaluation of which type of hoof protection provides the best traction on a given surface or whether barefoot hooves may even offer an advantage. To demonstrate the usefulness and practicality of the VGT, we tested it under four different hoof conditions (barefoot, a standard steel shoe, and two different glue-on shoes) on five different surfaces (sand, sand with additives/fiber at 25 °C and frozen at −18 °C, and turf). Our hypothesis is that the VGT provides reliable results and that glued-on horseshoes provide better grip on all surfaces compared to conventional horseshoes and bare hooves.

## 2. Materials and Methods

For this project, five different surfaces relevant to a horse’s performance in sport and safety in its daily living environment were tested: sand, frozen sand, sand with additives, frozen sand with additives, and turf. These tests were conducted for three different types of hoof protection (traditional iron shoes and two pairs of glue-on shoes, the second one functioning as a reference of a model without additional grip plates, provided by N&U Sport Horse Shoes GmbH) and the bare hoof without any additional protection.

The following abbreviations will be used in the article, see Table 1.

Measurements of rotational grip on the different surfaces were conducted at the facility of the University of Veterinary Medicine Vienna (VetMed Vienna). The tracks used to evaluate lameness in horses outside of the university’s riding arena were used as test grounds.

Before testing, all materials and surfaces used were assigned an ID and fitted onto the Vienna Grip Tester (VGT). In order to conduct the testing, an additional person was needed to properly use the equipment. This person also recorded the results of the measurements in a handwritten protocol, which was later digitalized and is provided as Table 2.

**Figure 1:**
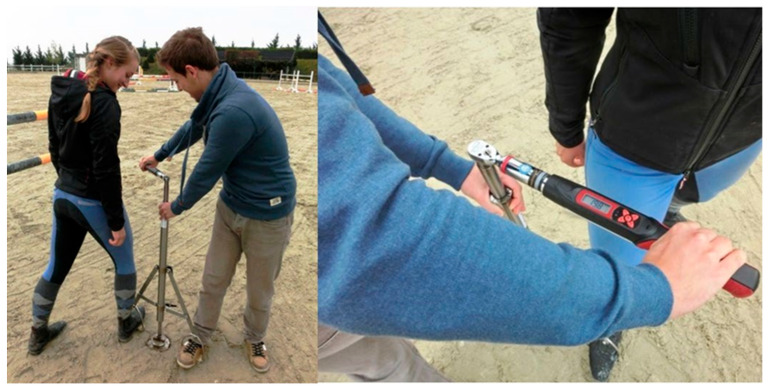
The device Vienna Grip Tester (VGT) under practical conditions. For further information, see https://www.youtube.com/watch?v=IvbQJomkL_8&t=32s (accessed on 25 January 2025).

The data was collected through the use of a torque wrench (DTW-100f, Checkline Europe, Bad Bentheim, Germany) fitted onto the VGT (Figure 1), which was provided by the Movement Science Group of VetMed University Vienna [6]. The maximum torque (rotational resistance) was measured on every surface in Nm. A minimum of 11 and a maximum of 19 measurements with a load of approximately 700 N were taken per ground and hoof protection combination, resulting in 305 measurements, which were later digitalized (see Table 2). The turf data were taken from the master’s thesis of Zinkanel [7]. The spring used in this Grip Tester is the Monroe (Grand Rapids, MI, USA) (ML5653), which has an extension force of 550 N on its own. With the added weight of the entire apparatus, the total force amounts to approximately 650 to 700 N [6,7]. The VGT is described in detail in Patent EP3141886A1 [6].

### 2.1. Surfaces

**Figure 2:**
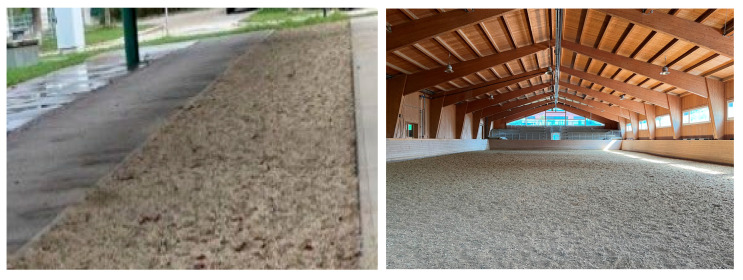
Test track for horses (**left**) and the indoor riding arena (**right**) on the university campus.

**Figure 3:**
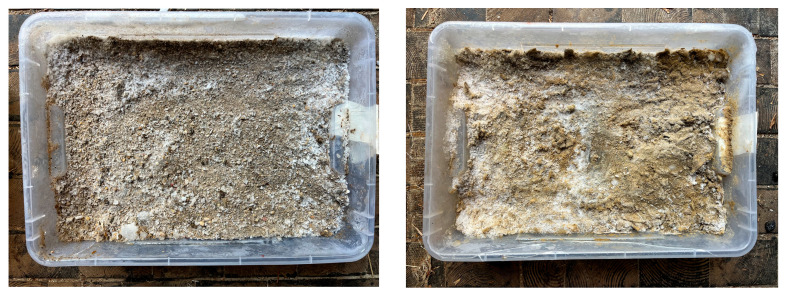
The boxes (40 cm × 30 cm × 10 cm) with frozen sand (SDF, **left**) and frozen sand with additives (SDAF, **right**).

Either the bare hoof or the iron horseshoe was mounted on the device. The glue on the shoes were fixed on the iron shoe with three screws. The spring stroke was adjusted so that the same force was applied under all conditions (Figure 1). The surfaces of the test track and the indoor riding arena were measured (Figure 2). Boxes were used to test the two different frozen sand surfaces (Figure 3). The box height of 20 cm met the standard criteria for a footing layer of 8–15 cm, ensuring realistic layering conditions.

Two people loaded the VGT spring by stepping into the stirrups to apply a normal force of 700 N to the tested surface–hoof combination (Figure 1). The selected load of 700 N is within a range that can be easily applied by two people and is sufficient to adequately compress the surface. One of the test persons rotated the torque wrench with a maximum holding function by 45 degrees. This process was repeated several times at different locations.

The torque can be explained by the normal force applied by the spring, the moment arm, and friction coefficient (see following equation) [6,7,10].

Ms = r × N × µs;(1)

Here, Ms—torque; R—moment arm; N—normal force; µs—friction coefficient.

### 2.2. Hoof Variants

**Figure 4:**
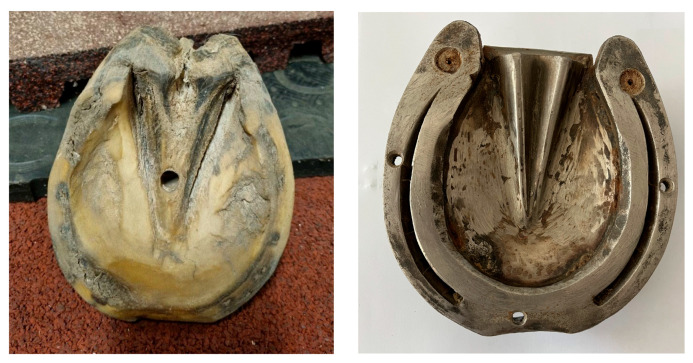
Natural bare hoof B (**left**) and the iron shoe I (**right**).

**Figure 5:**
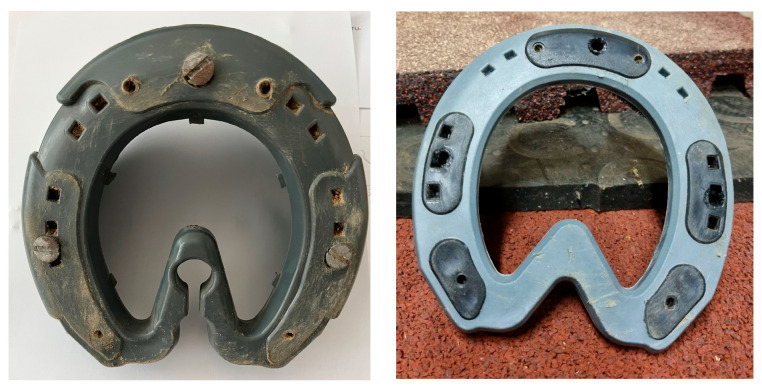
The glue-on shoes without (**left**, G) and with grip plates (**right**, GG).

Four types of hooves were used for this test series: a natural hoof provided by the Department of Anatomy without any additional protection (Figure 4), an iron horseshoe, and two glue-on variants provided by N&U Sport Horse Shoes GmbH (Figure 5). While conducting the measurements, the hoof and shoes were placed flat on the tested surface, and the Grip Tester was held at a 90-degree angle. The glue-on variants were applied to the iron shoe instead of the bare hoof to allow for easier fitting and removal. This did not influence their grip when conducting the measurements [2].

Each surface–shoe combination was tested at least 11 times. The *p*-value was taken into consideration to interpret the significance of differences between the various hoof models.

### 2.3. Statistics

The software SPSS (27.0, IBM, Chicago, IL, USA) was used to compare the differences. Normal distribution of the data was checked with the Kolmogorov–Smirnov test. To compare the different groups (surfaces and hoof shoes), an ANOVA for repeated measures was used. The Bonferroni correction was used to adapt the multiple comparisons. The results of the tests were put into Microsoft EXCEL and later imported into SPSS for processing. The boxplots used were also made with SPSS, and tables displaying the data were taken from the SPSS statistics output.

## 3. Results

**Figure 6:**
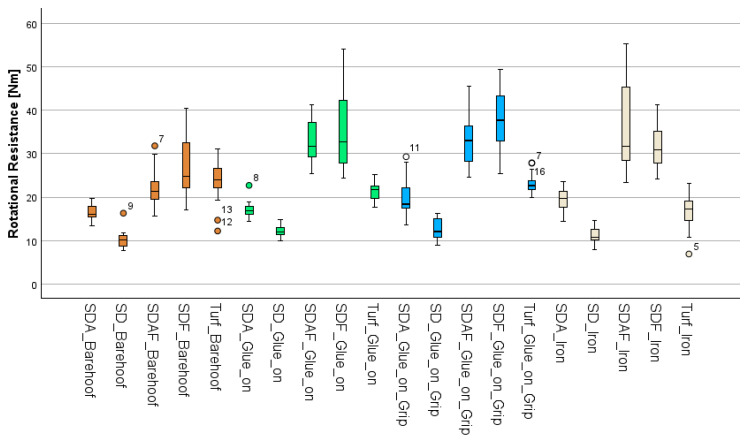
Grip, represented by the rotational resistance of all surface–hoof/shoe combinations. Freak values are marked with the numbers in the boxplot.

When comparing the different surfaces, a significant difference in grip was observed. SDA (18.3 ± 3.3 Nm) differed from all other surfaces (*p* < 0.001) except turf (21 ± 4.3 Nm, *p* = 0.36). Turf showed a significantly higher grip than sand (11.5 ± 2.2 Nm, *p* < 0.001) but less grip than the frozen surfaces SDAF (31 ± 8.9 Nm, *p* < 0.001) and SDF (33 ± 8.7 Nm, *p* < 0.001). SD, on the other hand, offered significantly lower rotational grip compared to all other surfaces (*p* < 0.001). The lowest grip on all surfaces was demonstrated by B (20.8 ± 7.2 Nm) and I (23 ± 11 Nm) compared to the G (24.8 ± 10.4 Nm) and GG (26.2 ± 10.3 Nm). There was no significant difference between B and I (*p* = 0.096), with B exhibiting significantly less grip than G (*p* = 0.015) and GG (*p* = 0.002), as well as I (G (*p* < 0.001) and GG (*p* < 0.001). GG and G did not differ significantly (*p* = 1). Table 3 shows all measured values, and Figure 6 shows a boxplot of all conditions.

## 4. Discussion

The present study investigated the rotational grip of 20 surface–hoof protection combinations using the Vienna Grip Tester (VGT), a newly developed device designed to quantify torsional resistance on equestrian surfaces. The significant differences observed between surface types and hoof protection variants underscore the importance of considering both components when aiming to enhance equine safety and performance [3,6,11,12].

The data clearly demonstrate that surface type has a substantial influence on rotational grip. As expected, frozen surfaces (SDAF and SDF) exhibited the highest torque values, likely due to increased surface rigidity, which reduces hoof displacement during rotation. Turf also provided relatively high grip values and did not differ significantly from sand with fiber (SDA), indicating that additive-enhanced surfaces can closely replicate the performance characteristics of natural turf. In contrast, dry sand (SD) showed the lowest grip values, highlighting the risk of slippage and potential injury on such loose substrates, particularly during high-speed equestrian activities [11,12,13].

With regard to hoof protection, the glue-on models (G and GG) consistently outperformed the traditional iron shoe (I) and barefoot condition (B). While differences between the glue-on variants were not statistically significant, both showed significantly higher grip values than B and I across multiple surfaces. These findings suggest that modern glue-on solutions offer a more favorable interaction with the ground by enhancing rotational stability, possibly due to material flexibility and improved contact surface geometry. This confirms earlier studies that emphasized the potential of alternative shoeing systems to reduce slip-related injuries in horses [3,6,13,14,15,16].

We were able to demonstrate that the VGT is a useful and practical tool for assessing the grip of ground–shoe combinations based on rotational resistance. Its design allows for standardized application of a vertical force of approximately 700 N, corresponding to a body mass of 70 kg [4]. With a total weight of only 9 kg, the VGT is portable and suitable for use in various field conditions, making it an accessible solution for researchers and practitioners alike [4,7,8].

The Vienna Grip Tester proved to be a reliable and reproducible method for assessing torsional grip. By applying a standardized normal force and measuring torque through a mechanical torque wrench, the VGT effectively simulates lateral hoof–ground interactions. Compared to traditional friction coefficient measurements, torsional resistance offers a more dynamic and relevant metric for equine movement, particularly during turning, jumping, and lateral maneuvers [4,6,7,8,11]. Although it is a quasi-static method, it has proven to be robust, as demonstrated by the consistent results and statistically significant outcomes.

One of the major strengths of this study lies in its practical setup. The use of anatomically correct hooves and real-world surfaces enhances ecological validity [2,8,17]. However, several limitations must be acknowledged. First, although mounting the glue-on shoes to iron shoes for testing facilitated standardized fitting, this setup may not fully replicate real-world conditions. Second, environmental factors such as moisture, wear, and temperature fluctuations were not systematically controlled beyond the frozen versus non-frozen comparison, which may affect grip dynamics. Additionally, the testing simulated static or quasi-static torque application, which may differ from the dynamic loading patterns seen in live animals [11,12,17].

Future research should explore the dynamic validity of the VGT under in-motion conditions, ideally by integrating biomechanical analysis or in vivo testing on live horses [7,11,12,13,17,18]. Furthermore, incorporating sensor-based real-time data acquisition (e.g., digital torque sensors, pressure mapping) could enhance precision and allow for continuous monitoring of hoof–surface interactions in training or competition environments [18].

A recent study investigated different testing devices for assessing rotational traction and demonstrated that the choice of methodology can substantially affect the outcomes. Two devices were compared on artificial turf, and the results showed that measurements strongly depended on the test configuration. Such conclusions are also relevant to the evaluation of equestrian surfaces, as they highlight that the reliability and interpretability of traction measurements can be significantly influenced by the device and protocol employed [19].

## 5. Conclusions

In summary, this study confirms the Vienna Grip Tester as a valid and effective sensor-based tool for assessing hoof–surface rotational grip. The results provide compelling evidence for the superiority of glue-on shoes in enhancing grip on a variety of performance surfaces, which has direct implications for equine safety, injury prevention, and sport optimization.

## Figures and Tables

**Table 1:** Abbreviations of the different surfaces used in the test.

ID	Surface	Description
SD	Sand	VetMed Track for checking soundness
SDF	Frozen sand	VetMed Track for checking soundness
SDA	Sand with additives	VetMed Track for checking soundness
SDAF	Frozen sand with additives	VetMed Track for checking soundness
T	Turf	Garden of the VetMed University

**Table 2:** All measurements of the bare hoof and the three hoof protectors on all surfaces. (orange: natural bare hoof, green: plastic glue-on shoe, blue: anti-slip glue-on shoe, gray: iron horseshoe).

SDA B	SD B	SDAF B	SDF B	Turf B	SDA G	SD G	SDAF G	SDF G	Turf G	SDA GG	SD GG	SDAF GG	SDF GG	Turf GG	SDA I	SD I	SDAF I	SDF I	Turf I
16	10.1	15.6	21.6	24	16.2	13.5	26.1	52.1	25	13.6	11.3	24.6	33	24	16.9	12.2	49.1	25.8	20
15.6	10.9	21.3	23	26	17.9	12	37.1	42.3	22	17.2	14.8	36.3	40.1	21	19.9	14.6	44.1	31.3	17
13.4	8.2	18.2	25.3	23	17.5	12.1	37.3	54.1	20	25.8	10.5	28.2	47.8	22	22.4	11	28.4	25.4	11
19.1	11	21.9	22.2	22	18.8	11.8	25.3	24.3	22	17.9	10.1	33.1	25.5	24	21.1	10.4	23.3	35.1	12
17.1	11.7	29.8	34.2	20	16.4	13	31.6	27.9	18	17.8	12.9	32.9	30.6	23	18.3	10.6	31.4	30.6	6.9
15.3	9.8	21.3	40.3	27	17	12.9	31.7	31.9	19	17.4	10.9	27.8	47.1	26	17.4	14.2	43.4	30.3	13
19.7	7.7	31.8	18.8	27	14.5	10.7	37.1	27.8	21	28.1	11.8	35.5	43.3	28	18.8	12.6	53.1	38.9	11
15.7	9.3	17.6	24.8	22	22.7	14.8	33.5	26.9	24	22.1	8.9	40.4	36.5	24	22	7.9	55.2	27.9	17
18.5	16.3	21.2	23.2	26	15.1	11.7	31.5	36	24	18	12.4	31.1	49.4	23	17.8	8.6	29.4	36.5	18
16.3	7.7	23.6	20.2	26	16.8	9.9	41.3	31	22	16.3	15.5	40.3	36.1	23	19.7	10.5	25.7	30.1	17
14.7	11.2	22.2	32.5	23	18.2	12.4	36.3	32.8	18	29.3	16.3	45.5	43.1	22	14.5	9.8	26.8	32.4	18
	10.2	25.2	25.3	12	16	11	29.3	38.2	25	19.9	15.2	29.8	41.9	24	21.4	12.7	31.8	41.3	19
		18.3	34.7	15	15.3		25.9	32	21	18.9		35.5	30.5	20	23.5		46.5	24.2	19
		23.7	24.2	28				50	22	19		28.2	27.3	24	16.2		38.7	32.9	18
		20.7	26.6	31				36.4	23				37.8	22	20.2		28.5		23
			40.4	23				27.3	22				37.8	28					16
			17.1	27				27.9	22				47.6	22					17
				19				46.9	18					21					20
				24				42.3	20					22					19
				27					20					23					19

**Table 3:** Abbreviations of the different test models.

ID	Hoof Application	Description
B	natural bare hoof	A natural bare hoof provided by the anatomy department of VetMed Vienna
G	plastic glue-on shoe	Provided by N&U Sport HorseShoes GmbH (Baden, Austria)
GG	anti-slip glue-on shoe	Provided by N&U Sport Horse Shoes GmbH, fitted with additional anti-slip plates
I	iron horseshoe	A commercial iron horseshoe fitted on a metal plate, provided by VetMed Vienna

## Data Availability

Data are available at the University of Veterinary Medicine and can be downloaded upon request.
